# Non‐invasive genomics of respiratory pathogens infecting wild great apes using hybridisation capture

**DOI:** 10.1111/irv.12984

**Published:** 2022-04-06

**Authors:** Livia V. Patrono, Caroline Röthemeier, Leonce Kouadio, Emmanuel Couacy‐Hymann, Roman M. Wittig, Sébastien Calvignac‐Spencer, Fabian H. Leendertz

**Affiliations:** ^1^ Project Group Epidemiology of Highly Pathogenic Microorganisms Robert Koch Institute Berlin Germany; ^2^ Department of Ecology and Emergence of Zoonotic Diseases Helmholtz Institute for One Health Greifswald Germany; ^3^ Laboratoire National d'Appui au Développement Agricole/Laboratoire Central de Pathologie Animale Bingerville Côte d'Ivoire; ^4^ Department of Primatology Max Planck Institute for Evolutionary Anthropology Leipzig Germany; ^5^ Taï Chimpanzee Project Centre Suisse de Recherches Scientifiques Abidjan Côte d'Ivoire

**Keywords:** non‐invasive health monitoring, pathogen genomics, respiratory disease, wild great apes

## Abstract

Human respiratory pathogens have repeatedly caused lethal outbreaks in wild great apes across Africa, leading to population declines. Nonetheless, our knowledge of potential genomic changes associated with pathogen introduction and spread at the human‐great ape interface remains sparse. Here, we made use of target enrichment coupled with next generation sequencing to non‐invasively investigate five outbreaks of human‐introduced respiratory disease in wild chimpanzees living in Taï National Park, Ivory Coast. By retrieving 34 complete viral genomes and three distinct constellations of pneumococcal virulence factors, we provide genomic insights into these spillover events and describe a framework for non‐invasive genomic surveillance in wildlife.

## 1 MAIN

Spillover of common human respiratory pathogens to wild great apes habituated to human presence for research or tourism has been repeatedly documented.^1^, ^2^, ^3^ The development of long‐term health monitoring programmes within conservation initiatives,^4^ bringing together behavioural observations and non‐invasive sampling (e.g. faeces, urine and performing necropsies on dead wildlife), has led to the establishment of a framework for studying disease epidemiology in these endangered populations. Viruses of different families have been identified as primary causative agents, with members of the family *Pneumoviridae* being frequently reported. Bacterial co‐infections, most often caused by *Streptococcus pneumoniae*,^5^, ^6^ have contributed to disease severity, ultimately leading to mortality.

Emergence of a pathogen in a new host population raises many questions, such as whether the pathogen will spread efficiently, whether mutations will arise and be fixed whilst spreading and whether it will remain endemic in the population or not. With the exception of a few recent reports,^2^, ^3^, ^7^ earlier molecular characterisations of the human pathogens causing disease in wild great apes have been limited to a few, partial genes. These investigations were conducted using PCR‐based screening approaches aimed at detecting and genotyping common respiratory agents infecting humans.^6^, ^8^ To better understand pathogen introduction, more comprehensive genomic analyses, ideally performed on samples collected at different stages of an outbreak and from different individuals, are required. Unbiased metagenomic/metatranscriptomic analyses may allow to assemble complete genomes,^2^, ^3^ but these are likely to require relatively deep sequencing when applied to samples with a low ratio of target (respiratory pathogen) versus background (host and other microorganisms) RNA/DNA, such as faeces. Implementing a target enrichment step prior to high throughput sequencing (HTS) can help overcome this limitation by focusing sequencing efforts on the RNA/DNA of interest,^7^ thus drastically reducing the sequencing depth required. Here, we made use of hybridisation capture coupled to HTS to re‐analyse a set of necropsy and faecal samples collected from wild chimpanzees, aiming at generating complete genomes from human pneumoviruses and characterising virulence proteins of pneumococci that caused outbreaks in the early 2000s.

Between 2004 and 2009 we monitored six outbreaks of respiratory disease in two communities (South and East) of wild, human habituated chimpanzees^9^ in Taï National Park, Ivory Coast^6^, ^8^, ^10^ (Table 1). Faeces were collected from as many individuals as possible and full necropsies were performed on the chimpanzees that succumbed to the infection. Initial PCR diagnostics performed on lung tissue of deceased chimpanzees,^1^ and at a later time on faeces of chimpanzees with clinical signs,^6^, ^8^ targeting partial glycoprotein (G) and phosphoprotein (P) viral genes allowed for the identification and genotyping of the causative agents. To start exploring potential genome‐wide diversity, we designed an RNA bait set encompassing complete human metapneumovirus (HMPV) and human orthopneumovirus (HRSV) type A and B genomes (synthesised by a service provider, MYBaits®, supporting information methods). We first validated the technique by performing two rounds of 24‐h hybrid capture on libraries built from 12 lung RNA extracts previously identified as positive for either pneumovirus via PCR (supporting information Table S1). On‐target reads represented from 0.1% to 99.08% of all reads and six complete viral genomes (three HMPV, two HRSVB and one HRSVA) could be reconstructed. To investigate whether we could broaden our dataset, we applied the method to 32 pneumovirus‐positive faecal samples collected during the different outbreaks and tested in previous studies.^6^, ^8^ Our sample selection aimed at maximising the number of individuals and the time frame tested. On‐target reads represented from 69.3% to 99.74% of all reads, and 21 complete viral genomes were reconstructed (28 at 2X coverage), highlighting a good performance also on non‐invasive samples (Table 1 and supporting information Table S1). As opposed to necropsy samples, which are collected (at the earliest) at the time of death several days after symptoms' onset, faeces can be collected continuously throughout the different phases of the infection, allowing to retrieve information from animals who survive (here, *n* = 27). This shows how despite not being the gold standard for respiratory viral infection diagnostics, faecal samples are well suited for and extend the reach of genomic studies of such pathogens in wildlife.

**TABLE 1:** Non‐invasive samples analysed via in‐solution hybridisation capture for viral enrichment

Outbreak period	Chimpanzee group	Virus identified	Lung samples tested	Faecal samples tested	Period covered	Complete genomes 20X (lungs, faeces)	Complete genomes 2X (lungs, faeces)
March 2004	South	HMPV^a^	3	12	08.03.2004 to 02.04.2004	7 (2, 5)	8 (1, 7)
October 2004	South	HMPV	n.a.	1	29.10.2004	0	0
August 2005	South	HRSVB	n.a.	14	16–24.08.2005	14	‐
February 2006	South	HRSVB^a^	1	0	10.02.2006	1	‐
February 2006	East	HRSVB^a^	2	0	07–09.02.2006	1	1
November 2009	South	HRSVA^a^	6	5	27.11.2009 to 17.12.2009	5 (1, 4)	1 (0, 1)

*Note*: Shown is a summary of the necropsy and faecal samples selected for viral enrichment and the respective results of genome coverage depth.

Abbreviations: HMPV, human metapneumovirus; HRSVB, human orthopneumovirus.

^a^ Co‐infection with *Streptococcus pneumoniae*.

In all samples analysed from each outbreak, viral genomes identified in different chimpanzees and over time were identical. By relaxing the consensus calling criteria (from at least 20 reads and 95% agreement to at least two reads and 65% agreement), one or two single nucleotide polymorphisms (SNPs) emerged in all outbreaks studied (supporting information Tables S2 and S3), which is in line with what is generally observed in within‐household and nosocomial transmission of pneumoviruses in humans.^11^ Further to suggesting that these spillovers likely represent single introduction events, these data point towards an efficient replication and spread without the insurgence of adaptive mutations.

Previous analyses based on a partial fragment of the G gene of the HRSVB had suggested that the virus causing the 2005 and 2006 outbreaks in the South group was identical.^8^ Our genome‐wide analyses revealed the existence of nine SNPs distributed over five genes that distinguish the two viruses (supporting information Table S2). This highlights the finer resolution allowed for by genome‐wide analyses and confirms that, as observed in humans, an infection with HRSV does not provide protection from re‐infection with a highly similar, homologous subtype, even when occurring just 6 months apart. To investigate whether the same was true for HMPV, we compared positive faecal samples from the only individual in which HMPV infection was confirmed in both March and October 2004. Despite retrieving only 37.5% of the viral genome from the October sample, we could assess that this portion was identical to the strain that circulated 7 months earlier. The evidence of re‐infection with seasonal human strains stresses even more the threat that these viruses pose to remaining great ape populations and the importance of implementing strict hygiene rules at tourism and research sites.^4^

Whole‐genome maximum‐likelihood phylogenetic analyses confirmed previous genotyping, with the 2004 HMPV strain falling within the diversity of the B2 lineage (supporting information Figure S1), the HRSVB from 2005 and 2006 within the GB3 genotype (supporting information Figure S2) and the HRSVA from 2009 within the GA2 (supporting information Figure S3). We acknowledge that given the paucity of genomic information available for viral strains circulating in humans in these remote areas, we could not fit these data to seasonal local patterns.

In four out of six outbreaks, several deaths occurred among the chimpanzees and were attributed to co‐infections with *S. pneumoniae* (pneumococcus). The availability of necropsy samples allowed for an initial characterisation of pneumococcal strains via Multi Locus Sequence Typing (MLST) and serotyping. These analyses identified three distinct serotypes^5^, ^6^ (ST 2309 in South 2004/2006, ST 2308 in East 2006, ST 8485 in South 2009), one of which was of unequivocal human origin (ST 8485). Bacterial isolation was successful only for the latter,^6^ highlighting the need of alternative tools to characterise more in depth pneumococci threatening wild populations.

To broaden the efficacy of the available polyvalent conjugate vaccines directed against pneumococcal capsular polysaccharides, recent vaccine development has been focused on targeting one or more virulence proteins^12^ such as choline binding proteins (e.g. CbpA, PspA, PcpA), serine‐rich repeat proteins (Psrp), pneumococcal pili (RrgA, B and C) favouring adherence and surface enzymes like the hyaluronate lyase (HysA), which favours tissue invasion. Presence and genetic variability of these proteins are known to vary substantially across serotypes identified in humans.^13^ To which extent the same occurs in pneumococcal strains circulating in wildlife remains largely unknown. To investigate the diversity of virulence factors identified in the pneumococci infecting the Taï chimpanzee population and to simultaneously design a tool that would allow for the differentiation of *S. pneumoniae* from other commensal streptococci (e.g. *Streptococcus. mitis* and *Streptococcus oralis*), we designed a bait set targeting nine (entire or partial) virulence genes thus far only reported in pneumococci (supporting information Table S4). We generated DNA baits by using sheared long‐range PCR products to which biotinylated adapters were subsequently attached.

Following hybrid capture on libraries generated from lung samples, 1.01% to 50.19% of the total reads were on target (supporting information Table S5), allowing for the characterisation of the virulence factors tested in the different strains. In the 2004 and 2006 outbreak samples from the South community, only five (CbpG, CbpA, PspA, HysA and PcpA) of the nine virulence genes tested were detected (supporting information Figure S4). When compared, consensus sequences for these genes were identical, suggesting the same strain was involved in both outbreaks. The same five virulence genes, plus a sixth one (Psrp), were detected in the 2006 East outbreak samples. Sequences, however, differed from those of the 2004/2006 South strain for all genes but HysA, which was identical in its full length (approximately 3 kb). The pneumococcal strain found in South in 2009 had yet another constellation, carrying all nine virulence genes tested. Sequences of the genes shared with the 2004/2006 South and the 2006 East strains differed from each other, in line with the previous (serotype) identification of three distinct strains. The greatest diversity was recorded in the choline binding (CbpA and PspA) and serine‐rich repetitive (Psrp) proteins (up to 33%, supporting information Figure S4), which are known to be highly polymorphic.^14^ From a functional perspective, the hyaluronidase gene of the 2004 and 2006 strains displayed an early truncation due to a one base pair deletion, suggesting potential functional loss. Inactivity of this enzyme due to indels or mutations in the coding gene has been reported for other virulent streptococci,^15^ implying that this gene is not an essential virulence factor.

To test the suitability of the method to faeces, we performed hybridisation capture on three libraries built from faecal extracts of the 2009 outbreak. On‐target reads represented 6.27% to 12.38% of all reads, falling within the range of what was observed for the lungs (supporting information Table S5). Similarly, the profile of virulence factors was comparable, with all nine genes being detected and identical sequences. Overall, gene profiling via hybridisation capture added layers of information on pneumococci responsible for mortality in wild chimpanzees, providing a framework for better understanding pathogenesis and tailoring potential emergency interventions (e.g. vaccinations). The power of this tool ultimately lies in the flexibility of simultaneously being able to study multiple aspects that may be relevant to species conservation, for example, adding baits to characterise genes associated with antibiotic resistance.

The results herein reported provide yet another example of the quality and quantity of information that can be obtained from non‐invasively monitoring outbreaks of disease in great apes. This should encourage the implementation of similar continuous surveillance programmes at other research sites or at least sampling faeces in the presence of clinical signs. To better understand the links between human and animal health, these should be coupled with similar studies in the local human population. Such comparative data could provide baseline evidence to guide improvements of wildlife conservation measures and local public health at once, for example, targeted vaccination campaigns for staff or local communities living around great ape habitats, following a truly One Health approach.

## CONFLICT OF INTEREST

No potential conflict of interest was reported by the authors.

## AUTHOR CONTRIBUTIONS

**Livia Victoria Patrono:** Conceptualization; formal analysis; investigation; methodology; project administration. **Caroline Röthemeier:** Investigation; methodology. **Leonce Kouadio:** Investigation; resources. **Emmanuel Couacy‐Hymann:** Resources; supervision. **Roman M. Wittig:** Funding acquisition; resources. **Sébastien Calvignac‐Spencer:** Conceptualization; methodology; supervision; validation. **Fabian H. Leendertz:** Conceptualization; funding acquisition; methodology; resources; supervision.

### PEER REVIEW

The peer review history for this article is available at https://publons.com/publon/10.1111/irv.12984.

## Supporting information


**Figure S1.** **Global HMPV phylogeny based maximum‐likelihood analyses performed on whole‐genome alignments.** Coloured dots represent sequences sampled from different continents. Sequences sampled from the Taï chimpanzees are in orange and fall within the B2 genotype. Scale bar is expressed in substitution per variable sites. Inner branch colours represent branch support values (grey is ≤0.95; black is >0.95).

**Figure S2.** **Global HRSVB phylogeny based on maximum‐likelihood analyses performed on whole‐genome alignments.** Coloured dots represents sequence sampled from different continents. Sequences sampled from the Taï chimpanzees are in orange (2005) and lilac (2006). Both fall within the GB3 genotype. Scale bar is expressed in substitution per variable sites. Inner branch colours represent branch support values (grey is ≤0.95; black is >0.95).

**Figure S3.** **Global HRSVA phylogeny based on maximum‐likelihood analyses performed on whole‐genome alignments.** Coloured dots represents sequence sampled from different continents. Sequences sampled from the Taï chimpanzees are in orange and fall within the GA2 genotype. Scale bar is expressed in substitution per variable sites. Inner branch colours represent branch support values (grey is ≤0.95; black is >0.95).

**Figure S4.** **Virulence gene profile observed in the three** ***Streptococcus pneumoniae*** **strains involved in the different respiratory disease outbreaks in the Taï chimpanzees.** Different colours correspond to different sequences for the gene of interest. White squares indicate that the gene was not detected. Numbers indicate % diversity with the strain below (e.g. 20 in ST 2309 Cbpa means 20% diversity between ST 2309 and ST 2308) except for numbers on the ST 8485 strain which indicate % diversity with ST 2309.

**Table S1.** Lung and faecal samples (indicated by an F preceding the number) analysed from the different respiratory outbreaks in the Taï chimpanzees. *these samples were collected during the HRSB 2006 outbreak in the East group, all others belong to the South group. n.a.: not available, indicates that there was not enough extract material left to repeat viral quantification prior to the capture experiment, therefore samples were included based on previous PCR results performed for the studies that initially reported these outbreaks (see references 8, 10 and 11 in the main manuscript). § in these samples sequence bleed‐through was detected and the results were not used for analysis. References used for mapping are KC562244 (~13 kb), KP317933 (~15 kb) and KP258739 (~15 kb).
**Table S2**. Nucleotide (nt) and amino acid (aa) changes observed in HRSV viruses responsible for the 2005 and 2006 outbreaks in the South group, and the 2006 outbreak in the East group. Positions correspond to reference genome KP258739.
**Table S3.** Nucleotide (nt) and amino acid (aa) changes observed in the HMPV virus responsible for the 2004 outbreak in the South group. Positions correspond to reference genome KC403971.
**Table S4.** Genes of interest and primers used to generate the *Streptococcus pneumoniae* amplicons. To amplify the hyaluronidase gene (HysA) two sets of partially overlapping primers were used.
**Table S5.** Lung and faecal samples (indicated by an F preceding the number) analysed for pneumococcal virulence factors from the different respiratory outbreaks in the Taï chimpanzees. *these samples were collected during the HRSB 2006 outbreak in the East group, all others belong to the South group. A concatenate of the genes of interest (each separated by a stretch of 100 Ns) was used as reference for mapping.

## Data Availability

Raw data are available in the European Nucleotide Archive, Project number PRJEB48718. Alignments of full viral genomes and pneumococcal genes are available in Zenodo (doi:10.5281/zenodo.5702462).
