# Supplementary figures and images for: Non‐invasive genomics of respiratory pathogens infecting wild great apes using hybridisation capture
Source: Influenza Other Respir Viruses. 2022 Apr 6;16(5):858–61. doi: 10.1111/irv.12984 (PMC9343332; doi:10.1111/irv.12984)

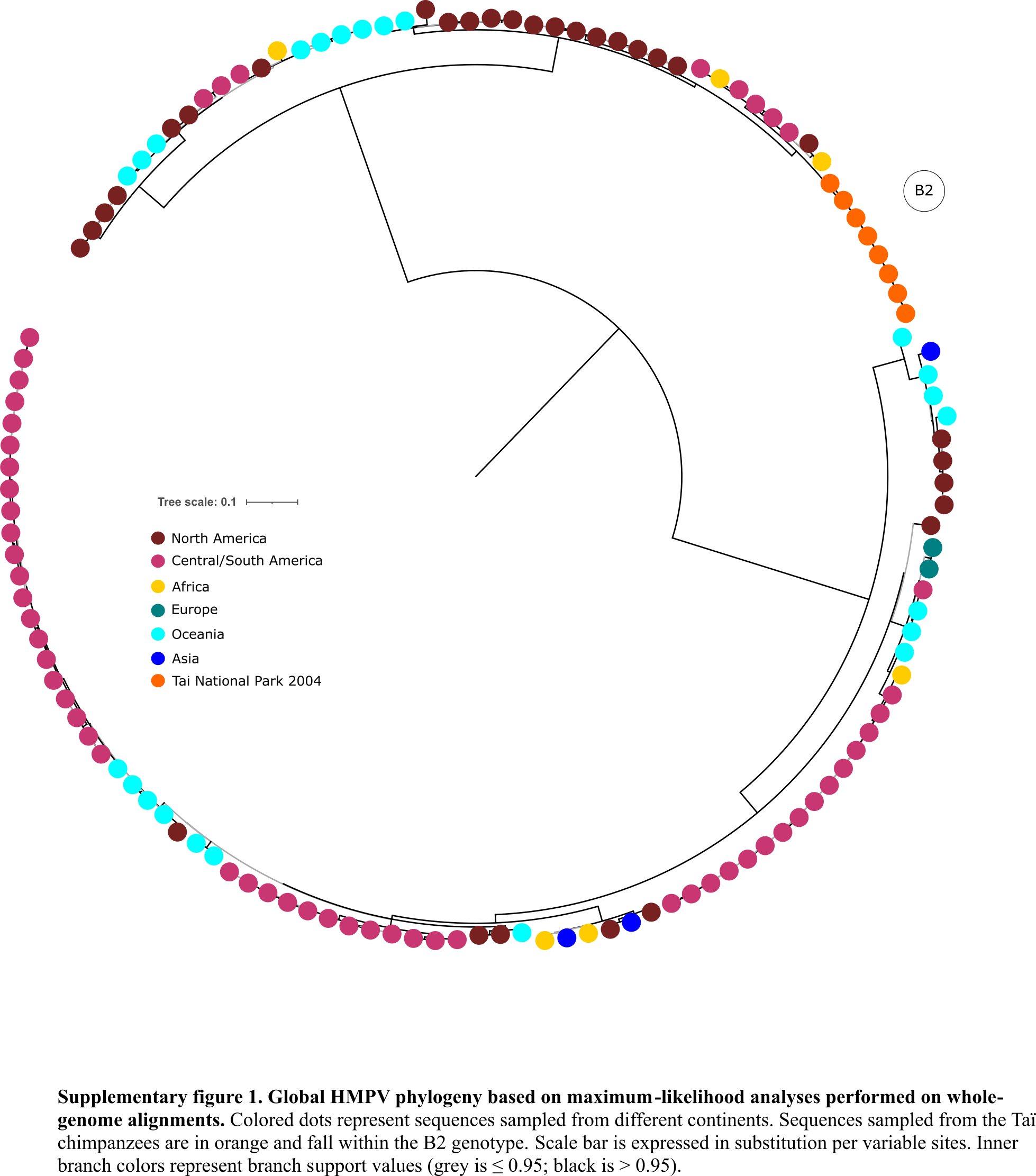

Supplement: Supplementary file 1 — Figure S1. Global HMPV phylogeny based maximum‐likelihood analyses performed on whole‐genome alignments. Coloured dots represent sequences sampled from different continents. Sequences sampled from the Taï chimpanzees are in orange and fall within the B2 genotype. Scale bar is expressed in substitution per variable sites. Inner branch colours represent branch support values (grey is ≤0.95; black is >0.95). [file IRV-16-858-s002.png]

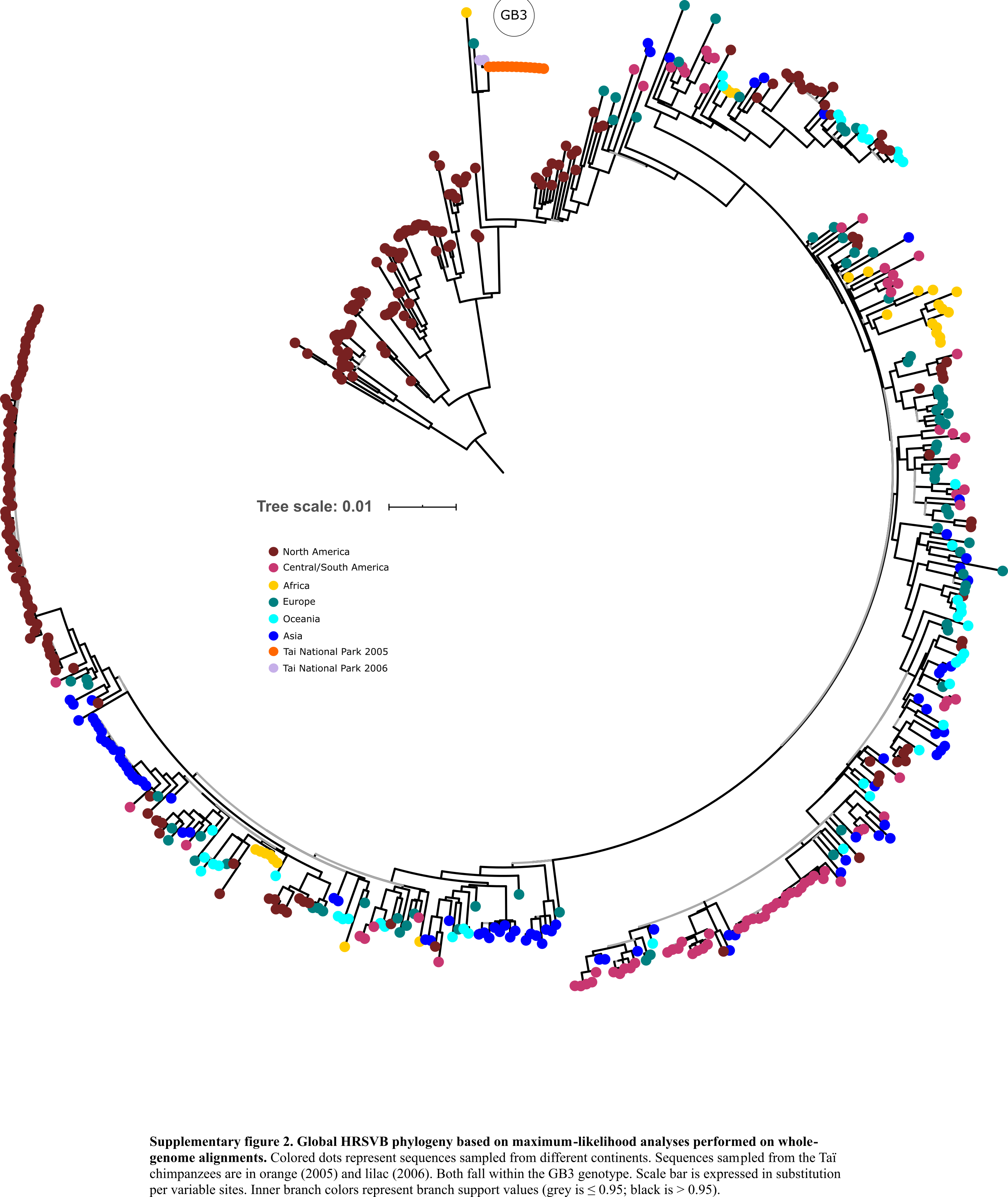

Supplement: Supplementary file 2 — Figure S2. Global HRSVB phylogeny based on maximum‐likelihood analyses performed on whole‐genome alignments. Coloured dots represents sequence sampled from different continents. Sequences sampled from the Taï chimpanzees are in orange (2005) and lilac (2006). Both fall within the GB3 genotype. Scale bar is expressed in substitution per variable sites. Inner branch colours represent branch support values (grey is ≤0.95; black is >0.95). [file IRV-16-858-s003.png]

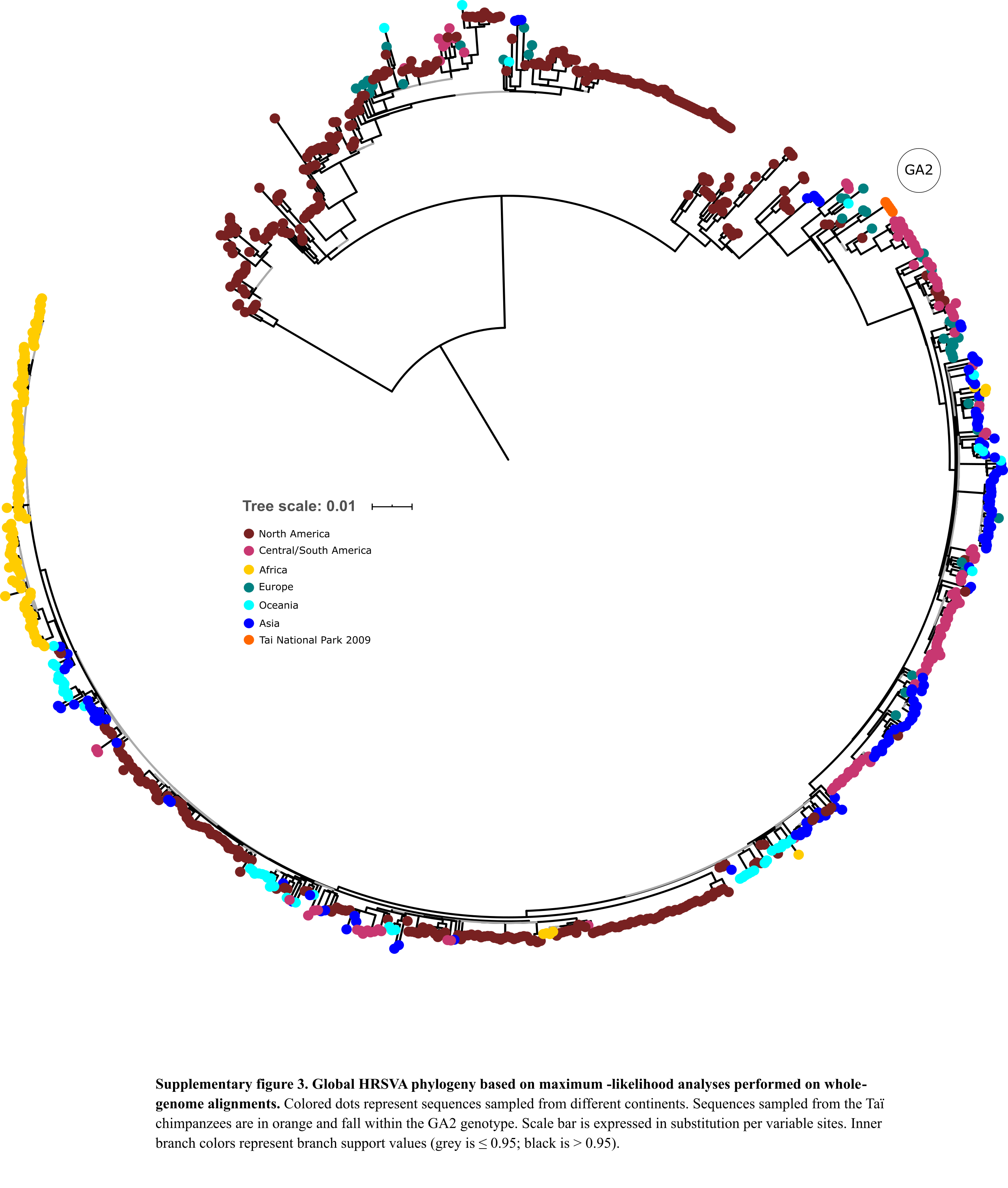

Supplement: Supplementary file 3 — Figure S3. Global HRSVA phylogeny based on maximum‐likelihood analyses performed on whole‐genome alignments. Coloured dots represents sequence sampled from different continents. Sequences sampled from the Taï chimpanzees are in orange and fall within the GA2 genotype. Scale bar is expressed in substitution per variable sites. Inner branch colours represent branch support values (grey is ≤0.95; black is >0.95). [file IRV-16-858-s004.png]

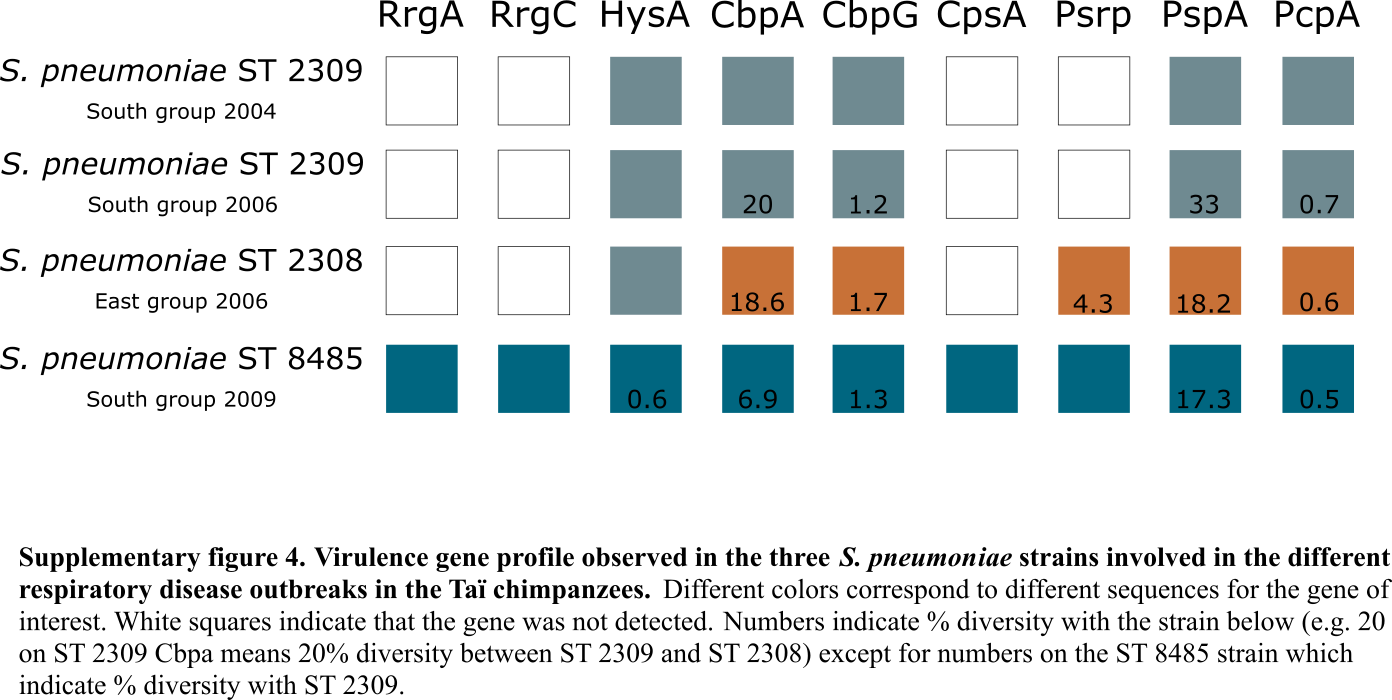

Supplement: Supplementary file 4 — Figure S4. Virulence gene profile observed in the three Streptococcus pneumoniae strains involved in the different respiratory disease outbreaks in the Taï chimpanzees. Different colours correspond to different sequences for the gene of interest. White squares indicate that the gene was not detected. Numbers indicate % diversity with the strain below (e.g. 20 in ST 2309 Cbpa means 20% diversity between ST 2309 and ST 2308) except for numbers on the ST 8485 strain which indicate % diversity with ST 2309. [file IRV-16-858-s001.png]
