# Supplementary material for: Non‐invasive genomics of respiratory pathogens infecting wild great apes using hybridisation capture
Source: Influenza Other Respir Viruses. 2022 Apr 6;16(5):858–61. doi: 10.1111/irv.12984 (PMC9343332; doi:10.1111/irv.12984)
Supplement: Supplementary file 5 — Table S1. Lung and faecal samples (indicated by an F preceding the number) analysed from the different respiratory outbreaks in the Taï chimpanzees. *these samples were collected during the HRSB 2006 outbreak in the East group, all others belong to the South group. n.a.: not available, indicates that there was not enough extract material left to repeat viral quantification prior to the capture experiment, therefore samples were included based on previous PCR results performed for the studies that initially reported these outbreaks (see references 8, 10 and 11 in the main manuscript). § in these samples sequence bleed‐through was detected and the results were not used for analysis. References used for mapping are KC562244 (~13 kb), KP317933 (~15 kb) and KP258739 (~15 kb). Table S2. Nucleotide (nt) and amino acid (aa) changes observed in HRSV viruses responsible for the 2005 and 2006 outbreaks in the South group, and the 2006 outbreak in the East group. Positions correspond to reference genome KP258739. Table S3. Nucleotide (nt) and amino acid (aa) changes observed in the HMPV virus responsible for the 2004 outbreak in the South group. Positions correspond to reference genome KC403971. Table S4. Genes of interest and primers used to generate the Streptococcus pneumoniae amplicons. To amplify the hyaluronidase gene (HysA) two sets of partially overlapping primers were used. Table S5. Lung and faecal samples (indicated by an F preceding the number) analysed for pneumococcal virulence factors from the different respiratory outbreaks in the Taï chimpanzees. *these samples were collected during the HRSB 2006 outbreak in the East group, all others belong to the South group. A concatenate of the genes of interest (each separated by a stretch of 100 Ns) was used as reference for mapping. [file IRV-16-858-s005.docx]

**Supplemental material for**

**Non-invasive genomics of respiratory pathogens infecting wild great apes using hybridization capture**

Livia V. Patrono, Caroline Röthemeier, Leonce Kouadio, Emmanuel Couacy-Hymann, Roman M. Wittig, Sébastien Calvignac-Spencer and Fabian H. Leendertz.

**Supplementary tables and figures**

| Sample ID | Individual | Outbreak | Collection date | Viral copy number in 5 µl of extract | % on-target reads | Positions in the reference genome covered 1x | Positions in the reference genome covered 20x |
| --- | --- | --- | --- | --- | --- | --- | --- |
| F209.1 | Besar | HMPV 2004 | 12.03.2004 | 3,02E+01 | 91.65 | 13065 | 9369 |
| F15.7 | Gogol | HMPV 2004 | 14.03.2004 | 4,99E+02 | 99.68 | 13243 | 13224 |
| F17.2 | Isha | HMPV 2004 | 08.03.2004 | 1,83E+02 | 99.74 | 13243 | 13243 |
| F21.7 | Kaos | HMPV 2004 | 14.03.2004 | 1,64E+02 | 93.68 | 12084 | 2425 |
| F22.5 | Kinshasa | HMPV 2004 | 14.03.2004 | 8,64E+01 | 99.01 | 13220 | 13174 |
| F20.5 | Julia | HMPV 2004 | 02.04.2004 | 2,23E+01 | 94.70 | 13239 | 11786 |
| F24.4 | Louise | HMPV 2004 | 12.03.2004 | 5,98E+02 | 99.47 | 13243 | 13242 |
| F27.3 | Romario | HMPV 2004 | 20.03.2004 | 3,49E+01 | 80.29 | 12723 | 7259 |
| F28.5 | Rubra | HMPV 2004 | 20.03.2004 | 2,59E+01 | 88.36 | 12543 | 7430 |
| F29.17 | Sagu | HMPV 2004 | 29.10.2004 | 4,08E+01 | 79.73 | 4201 | 2431 |
| F29.8 | Sagu | HMPV 2004 | 14.03.2004 | 1,40E+02 | 94.59 | 13238 | 8590 |
| F115.2 | Yucca | HMPV 2004 | 15.03.2004 | 2,10E+01 | 88.61 | 13018 | 11165 |
| F59.5 | Zyon | HMPV 2004 | 12.03.2004 | 1,17E+01 | 98.16 | 13242 | 13087 |
| 20.19 | Virunga | HMPV 2004 | 19.03.2004 | n.a | 4.16 | 13127 | 3428 |
| 21.17 | Ophelia | HMPV 2004 | 10.03.2004 | 8,64E+04 | 99.08 | 13239 | 13238 |
| 22.13 | Orest | HMPV 2004 | 10.03.2004 | 7,40E+01 | 91.33 | 13239 | 13238 |
| F15.5 | Gogol | HRSVB 2005 | 17.08.2005 | 3,11E+03 | 96.76 | 15101 | 14723 |
| F151.1 | Jacobo | HRSVB 2005 | 21.08.2005 | 1,58E+03 | 99.43 | 15101 | 15037 |
| F21.3 | Kaos | HRSVB 2005 | 18.08.2005 | 2,39E+03 | 99.62 | 15101 | 15101 |
| F22.4 | Kinshasa | HRSVB 2005 | 16.08.2005 | 2,02E+03 | 99.51 | 15101 | 15085 |
| F23.2 | Kuba | HRSVB 2005 | 16.08.2005 | 8,72E+02 | 99.55 | 15101 | 15097 |
| F24.2 | Louise | HRSVB 2005 | 16.08.2005 | 2,53E+03 | 99.61 | 15101 | 15101 |
| F50.4 | Sumatra | HRSVB 2005 | 17.08.2005 | n.a. | 99.32 | 15101 | 14833 |
| F50.6 | Sumatra | HRSVB 2005 | 24.08.2005 | n.a. | 98.80 | 15101 | 15020 |
| F51.3 | Taboo | HRSVB 2005 | 17.08.2005 | n.a. | 99.55 | 15101 | 15100 |
| F52.2 | Utan | HRSVB 2005 | 16.08.2005 | n.a. | 98.69 | 15101 | 14708 |
| F57.2 | Woodstock | HRSVB 2005 | 16.08.2005 | n.a. | 97.91 | 15101 | 14726 |
| F147.1 | Yucca | HRSVB 2005 | 18.08.2005 | n.a. | 98.58 | 15101 | 15063 |
| F58.2 | Zora | HRSVB 2005 | 16.08.2005 | n.a. | 98.74 | 15101 | 15029 |
| F59.2 | Zyon | HRSVB 2005 | 16.08.2005 | n.a. | 99.67 | 15101 | 15101 |
| 76.12 | Candy | HRSVB 2006* | 07.02.2006 | n.a. | 92.02 | 14849 | 8418 |
| 77.8 | Isha's baby | HRSVB 2006 | 10.02.2006 | 6,90E+02 | 98.32 | 15101 | 15101 |
| 78.9 | Vasco | HRSVB 2006* | 09.02.2006 | 3,90E+01 | 99.93 | 15101 | 15101 |
| 429.7 | Atra | HRSVA 2009 | 08.12.2009 | n.a. | 62.15^§^ | 13789 | 11357 |
| 431.5 | Wapi | HRSVA 2009 | 17.12.2009 | n.a. | 1.13^§^ | 13706 | 2711 |
| 557.9 | Olivia | HRSVA 2009 | 07.12.2009 | n.a. | 68.97 | 3129 | 400 |
| 558.11 | Louise | HRSVA 2009 | 07.12.2009 | n.a. | 0.1 | 1813 | 0 |
| 559.7 | Akrouba | HRSVA 2009 | 11.12.2009 | n.a. | 1.28^§^ | 12022 | 32 |
| 560.7 | Akwaba | HRSVA 2009 | 02.12.2009 | 5,00E+02 | 94.70 | 14216 | 14214 |
| F24.17 | Louise | HRSVA 2009 | 28.11.2009 | 4,50E+01 | 99.20 | 15018 | 14756 |
| F26.13 | Olivia | HRSVA 2009 | 29.11.2009 | 7,50E+01 | 69.30 | 15077 | 14780 |
| F26.11 | Olivia | HRSVA 2009 | 06.12.2009 | 6,80E+01 | 98.64 | 14661 | 11531 |
| F294.1 | Sassandra | HRSVA 2009 | 27.11.2009 | 9,10E+01 | 98.17 | 15039 | 14205 |
| F50.15 | Sumatra | HRSVA 2009 | 07.12.2009 | n.a. | 98.87 | 15146 | 15122 |

**Supplementary table 1:** Lung and faecal samples (indicated by an F preceding the number) analysed from the different respiratory outbreaks in the Taï chimpanzees. *these samples were collected during the HRSB 2006 outbreak in the East group, all others belong to the South group. n.a.: not available, indicates that there was not enough extract material left to repeat viral quantification prior to the capture experiment, therefore samples were included based on previous PCR results performed for the studies that initially reported these outbreaks (see references 8, 10 and 11 in the main manuscript). § in these samples sequence bleed-through was detected and the results were not used for analysis. References used for mapping are KC562244 (~13kb), KP317933 (~15kb) and KP258739 (~15kb).

| Sample ID | Gene | Nt change | AA change |
| --- | --- | --- | --- |
| 76.12, 78.9, 77.8 | NS1 | G376A | M126I |
| 76.12, 78.9, 77.8 | NS2 | T175C | F59L |
| 76.12, 78.9, 77.8 | SH | A144G | 48 |
| 76.12, 78.9, 77.8 | SH | T153C | 51 |
| F52.2 | M (non coding) | T961C |  |
| 76.12, 78.9, 77.8 | M2 | G11A | R4K |
| 76.12, 78.9, 77.8 | M2 | 7A4C | E25A |
| 76.12, 78.9, 77.8 | M2 | A124G | N42D |
| 76.12, 78.9, 77.8 | L | C512A | T171K |
| 76.12, 78.9, 77.8 | L | T2088C | 696 |
| 76.12 | L | C2589T | 863 |
| F57.2 | L | T5511C | 1837 |
| F154.1 | L | T5511C | 1837 |

**Supplementary table 2**. Nucleotide (nt) and amino acid (aa) changes observed in HRSV viruses responsible for the 2005 and 2006 outbreaks in the South group, and the 2006 outbreak in the East group. Positions correspond to reference genome KP258739.

| Sample ID | Gene | Nt change | AA change |
| --- | --- | --- | --- |
| F209.1 | N | C696T | 232 |
| F27.3 | F | G30A | 10 |
| F59.5 | G | T270C | 90 |
| F27.3 | L | A1013G | D338G |
| F21.7 | L | C2043A | 681 stop codon |
| F21.7 | L | C2112G | 704 stop codon |
| F28.5 | L | C3376A | Q1126K |
| F27.3 | L | 3664 2 nt deletion | early truncation |

**Supplementary table 3.** Nucleotide (nt) and amino acid (aa) changes observed in the HMPV virus responsible for the 2004 outbreak in the South group. Positions correspond to reference genome KC403971.

| **Gene** | **Forward Primer (5’🡪3’)** | **Reverse Primer (5’🡪3’)** | **Amplicon size (bp)** |
| --- | --- | --- | --- |
| CpsA | TCGCTCTTTGCAGTACAGCA | ATCCATCCGACCTGTCCCTT | 1074 |
| RrgA | CCTGAAACCAGTCCAGCGAT | GTCGCGCACTGTGTTCTTTT | 2016 |
| RrgC | AGCGCAAGAAGATCACACGT | ATCAATCCGTGGTCGCTTGT | 1015 |
| CbpG | GGGAGGGGAAGTGCGAATTT | CCACTCACCAGATGAAGCGA | 804 |
| LytA | ACAGATTTGCCTCAAGTCGG | ATCTGGCTCKACTGTRAATTCTG | 909 |
| HysA1 | GTGGTCAGCTTGGGTAGACC | CCCTGACTGTTCGAACGACA | 932 |
| HysA2 | TGTCGTTCGAACAGTCAGGG | AACGCCCCAAGTAGCAAGAA | 2028 |
| PcpA | CAAAGGCAGTCAGCTGGAGA | ACCCAACCAGTAGCCATTGA | 1014 |
| Psrp | TCAGCTTCAGAGTCGGCAAG | AGGCGCTTTGACTAGCACTT | 952 |
| PspA | AAACAAGAAAAYGGTATGTGGTACT | CACCCAACCTGTTGCCATTG | 725 |
| CbpA | ACAAGAAAACGGTATGTGGTACT | ACAGTTGTGTTGACTGCAAGG | 1031 |

**Supplementary table 4.** Genes of interest and primers used to generate the *Streptococcus pneumoniae* amplicons. To amplify the hyaluronidase gene (HysA) two sets of partially overlapping primers were used.

| Sample ID | Individual | Outbreak | Pneumococcal copy number in 5 µl of extract | % on target reads | Positions in the reference sequence covered 1x | Positions in the reference sequence covered 20x |
| --- | --- | --- | --- | --- | --- | --- |
| 20.19 | Virunga | HMPV 2004 | 1,79E+05 | 9.45 | 9623 | 8440 |
| 21.17 | Ophelia | HMPV 2004 | 8,00E+04 | 7.08 | 9851 | 7904 |
| 22.13 | Orest | HMPV 2004 | 2,44E+05 | 13.90 | 15094 | 11722 |
| 76.12 | Candy | HRSVB 2006* | 5,38E+04 | 18.42 | 10297 | 9541 |
| 77.8 | Isha's baby | HRSVB 2006 | 8,60E+03 | 8.09 | 9826 | 8480 |
| 78.9 | Vasco | HRSVB 2006* | 1,85E+03 | 31.16 | 14478 | 9739 |
| 429.7 | Atra | HRSVA 2009 | 7,15E+05 | 18.04 | 15004 | 14131 |
| 431.5 | Wapi | HRSVA 2009 | 8,93E+03 | 50.19 | 16120 | 14796 |
| 559.7 | Olivia | HRSVA 2009 | 5,65E+03 | 37.61 | 16035 | 14811 |
| 557.9 | Louise | HRSVA 2009 | 1,85E+06 | 22.14 | 15025 | 14202 |
| 558.10 | Akrouba | HRSVA 2009 | 4,48E+06 | 49.70 | 15780 | 14586 |
| 560.7 | Akwaba | HRSVA 2009 | 1,07E+05 | 1.01 | 14335 | 11229 |
| F17.10 | Isha | HRSVA 2009 | 1,27E+04 | 6.27 | 14727 | 12836 |
| F27.7 | Romario | HRSVA 2009 | 3,97E+04 | 9.21 | 15069 | 12533 |
| F31.5 | Akrouba | HRSVA 2009 | 2,37E+04 | 12.38 | 15012 | 13691 |

**Supplementary table 5:** Lung and faecal samples (indicated by an F preceding the number) analysed for pneumococcal virulence factors from the different respiratory outbreaks in the Taï chimpanzees. *these samples were collected during the HRSB 2006 outbreak in the East group, all others belong to the South group. A concatenate of the genes of interest (each separated by a stretch of 100 Ns) was used as reference for mapping.

**Supplementary methods**

**Samples**

The long-term health monitoring program of the Taï Chimpanzee project includes continuous collection of faecal samples from adult, individually recognized wild-living chimpanzees. Faeces are collected in 2 ml cryotubes with the aid of a plastic spatula right after observing defecation. In outbreak circumstances, field assistants and researchers attempt collecting samples from as many individuals as possible, including both symptomatic and asymptomatic. The health monitoring program of the Taï Chimpanzee project includes wildlife mortality surveillance. To this end, necropsies are performed by a trained veterinarian on all wildlife found dead in the research area. Tissue samples of all inner organs are taken, as far as the state of carcass decomposition allows. Necropsies follow a standardized protocol, including use of full PPE (personal protective equipment) due to the occurrence of anthrax, ebola virus disease and monkeypox in the area. The necropsy site is subsequently decontaminated according to World Health Organization guidelines, involving burial of the carcass and incineration or disinfection with 10% formalin of all contaminated materials. In the field, samples are stored in liquid nitrogen and formalin and subsequently shipped on dry ice to the Robert Koch Institute for analyses.

**Nucleic acid extraction and double stranded cDNA synthesis**

Total nucleic acids were extracted from 20 mg of lung tissue and up to 60 mg of faeces using the Viral RNA Mini kit (Qiagen) and the GeneMATRIX Stool DNA Purification Kit (Roboklon), respectively. To focus on the RNA component, extracts were subject to DNase treatment using the TURBO DNA-free kit^TM^ (Ambion). Seventeen microliters (µl) of extract were mixed with 1 µl of DNase enzyme, 5 µl of buffer and 27 µl of nuclease free water and incubated at 37°C for 30 minutes (min). The enzyme was then inactivated by adding to the mix 5 µl of DNase inactivation reagent. The RNA was purified using the MinElute PCR Purification kit (Qiagen), eluted in 10 µl and transcribed into cDNA using the SuperScript IV reverse transcriptase (ThermoFisher). For this reaction, 8 µl of RNA were mixed with 1 µl of a 10 µM random hexamer and 1 µl of 10mM dNTPs, and incubated at 65°C for 5 min. Upon cooling on ice for 1 min, 4 µl of 5X SSIV buffer, 1 µl of DTT, 1 µl of RNAaseOUT, 1 µl of SSIV enzyme and 3 µl of DPEC-treated water were added. The mix was then incubated according to the manufacturer’s instructions: 23°C for 10 min, 50°C for 10 min and 85°C for 10 min. The resulting cDNA was double stranded by adding to the reaction 8 µl of NEBNext Second Strand Synthesis Reaction Buffer, 4 µl of Second Strand Synthesis Enzyme Mix and 48 µl of nuclease-free water (Second Strand Synthesis Module, New England Biolabs). Double stranded cDNA was purified using Ampure XP magnetic beads (Beckam-Coulter), eluted in 20 µl low EDTA TE buffer and quantified using the Qubit High Sensitivity kit.

**Next-generation sequencing (NGS) and hybridization capture**

Illumina-compatible dual-index libraries were generated from up to 300 ng of DNA or double-stranded cDNA. The same amount of chicken DNA was used as negative control during library building and capture. Samples were fragmented using a Covaris S220 Focused-ultrasonicator® in a volume of 130 µl low EDTA TE buffer using settings to generate a 400 bp fragment size (Intensity = 4, Duty cycle = 10%, Cycles per burst = 200, Treatment time = 55 s, Temperature = 7°C). Fragmented DNA/cDNA was concentrated using the MinElute PCR purification kit and eluted into 50 µl low EDTA TE buffer. Dual-index libraries were built using the NEBNext Ultra II kit according to the standard protocol. Where more than 50 ng were used as input, a 400 bp size selection was performed upon adapter ligation using Ampure XP magnetic beads. Final libraries were quantified using the KAPA HiFi library quantification kit and stored at -20 °C until further use. Libraries were subject to target enrichment via hybridization capture. For this purpose, libraries were pooled and concentrated using the MinElute PCR Purification kit (Qiagen) to a final volume of 10 µl. For viral enrichment, two rounds of 24h hybridization capture at 65°C were performed using 2-fold tiling 80-mer RNA baits (MYBaits®) designed to target the whole genome of pneumoviruses. The bait design was proposed by the service provider and validated by our team. The MYBaits® Sequence Enrichment for Targeted Sequencing protocol (Version 2.3.1) was followed, with the exception that only a fourth of the recommended bait quantity was used per capture round. For the pneumococcal capture, we selected 9 genes thus far reported only in pneumococci, or divergent enough from other closely related *Streptococcus* species^1^. These included the CpsA, CbpA, CbpG, PspA, PcpA, Psrp, HysA, RrgA, and RrgB genes. The lytA gene was also included in the bait set but the resulting data was discarded due to high presence of reads mapping to other commensal streptococci (e.g. *S. oralis*, *S. mitis* and *S. pseudopneumoniae*). Baits were generated using sheared long range PCR (LA Taq polymerase, Takara) products obtained using the *S. pneumoniae* strain (ST 8485) isolated in the 2009 outbreak following previously published protocols^2,3^. Primers used for each target gene are available in the supplementary information. Two rounds of hybridization capture were performed as previously described^2^. After each round of capture, the captured library pool was amplified using the KAPA Hot Start Library Amplification system to reach a minimum of 200 ng of library and quantified using the KAPA HiFi Library Quantification Kit. The enriched pool was diluted to 4nM and sequenced on a MiSeq platform using the V3 chemistry (2x300 bp).

**Data analysis**

Raw reads were pre-processed using Trimmomatic v0.36^4^, with the following settings: LEADING:30 TRAILING:30 SLIDINGWINDOW:4:40 MINLEN:40. Trimmed reads were mapped to a reference genome (accession number KC562244 for HMPV, KP317933 for HRSVA, and KP258739 for HRSVB) using BWA-MEM v0.7.15-r1140 ^5^. We sorted mapping files and removed duplicates using the SortSam and MarkDuplicates tools from Picard v1.113 (<http://broadinstitute.github.io/picard>). Consensus sequences were generated using Geneious v.11. Base calling was set to 20 reads and 95% agreement. Where coverage was lower, base calling was set to 2 reads. Consensus sequences were aligned using MAFFT v7^6^. The same pipeline was used for *S. pneumoniae* using a concatenate of the genes of interest as a reference for the mapping along with reference genomes LR216061 and AE560072 (Genbank accession numbers). Maps were individually checked in Geneious v.11 and mapping of reads was further improved by using the mapping tools available in the software. Additional reference sequences were used to improve consensus sequences for pneumococcal genes CbpA (CP050175) and PspA (KY446363 and KY446287). When comparing consensus sequences, ambiguous and unambiguous bases were manually checked. The inclusion of a negative control (chicken DNA) allowed us to set a 0.01 and 1% contamination threshold (which expresses the % of on-target reads in this sample, most likely due to sequence bleed-through) for the viral and bacterial capture experiments, respectively.

**Phylogenetic analyses**

Datasets comprising all available HRSVA, HRSVB and HMPV complete genomes were downloaded from the National Center for Biotechnology Information (NCBI) database. Sequences were aligned using MAFFT v7 and Gblocks was run under stringent settings in Seaview v4^7^ to select conserved blocks. The online tool FaBox v1.4^8^ was used to collapse the alignment to unique sequences. The complete datasets (including the sequences herein generated) comprised 914 sequences for HRSVA, 543 for HRSVB and 130 for HMPV. Identical sites and sites containing ambiguities (Ns) were stripped using Geneious, therefore only variable sites were included in the analysis (529 bases for HRSVA, 609 for HRSVB and 3677 for HMPV). RDP4^9^ was used to identify and remove potential recombinant fragments. Model selection and maximum-likelihood analyses were conducted using iqtree^10^ for SNP data. Branch support was assessed using the approximate likelihood ratio test^11^ (aLRT, 1000 replicates). The resulting tree was visualized and edited in iTOL^12^ (https://itol.embl.de/).

**Supplementary references**

1 Donati C, Hiller NL, Tettelin H *et al.* Structure and dynamics of the pan-genome of Streptococcus pneumoniae and closely related species. *Genome Biol* 2010; **11**: R107.

2 Maricic T, Whitten M, Pääbo S. Multiplexed DNA sequence capture of mitochondrial genomes using PCR products. *PLoS One* 2010; **5**: 9–13.

3 Penalba J V., Smith LL, Tonione MA *et al.* Sequence capture using PCR-generated probes: A cost-effective method of targeted high-throughput sequencing for nonmodel organisms. *Mol Ecol Resour* 2014; **14**: 1000–1010.

4 Bolger AM, Lohse M, Usadel B. Trimmomatic: A flexible trimmer for Illumina sequence data. *Bioinformatics* 2014; **30**: 2114–2120.

5 Li H, Durbin R. Fast and accurate short read alignment with Burrows-Wheeler transform. *Bioinformatics* 2009; **25**: 1754–60.

6 Katoh K, Standley DM. MAFFT multiple sequence alignment software version 7: Improvements in performance and usability. *Mol Biol Evol* 2013; **30**: 772–780.

7 Gouy M, Guindon S, Gascuel O. Sea view version 4: A multiplatform graphical user interface for sequence alignment and phylogenetic tree building. *Mol Biol Evol* 2010; **27**: 221–224.

8 Villesen P. FaBox: An online toolbox for FASTA sequences. *Mol Ecol Notes* 2007; **7**: 965–968.

9 Martin DP, Murrell B, Golden M, Khoosal A, Muhire B. RDP4: Detection and analysis of recombination patterns in virus genomes. *Virus Evol* 2015; **1**: 1–5.

10 Nguyen LT, Schmidt HA, Von Haeseler A, Minh BQ. IQ-TREE: A fast and effective stochastic algorithm for estimating maximum-likelihood phylogenies. *Mol Biol Evol* 2015; **32**: 268–274.

11 Guindon S, Dufayard JF, Lefort V, Anisimova M, Hordijk W GO. New algorithms and methods to estimate maximum-likelihood phylogenies: assessing the performance of PhyML 3.0. - PubMed - NCBI. *Syst Biol* 2010; **29**: 307–21.

12 Letunic I, Bork P. Interactive Tree Of Life (iTOL) v4: recent updates and new developments. *Nucleic Acids Res* 2019; **47**: W256–W259.
